# High On-Current Ge-Channel Heterojunction Tunnel Field-Effect Transistor Using Direct Band-to-Band Tunneling

**DOI:** 10.3390/mi10020077

**Published:** 2019-01-24

**Authors:** Garam Kim, Jaehong Lee, Jang Hyun Kim, Sangwan Kim

**Affiliations:** 1 Department of Electrical and Computer Engineering (ECE), Seoul National University, Gwanak 599, Gwanak-gu, Seoul 151-742, Korea; kgr2487@gmail.com (G.K.) jaehong2@gmail.com (J.L.); neuburg@naver.com (J.H.K.); 2 Department of Electrical and Computer Engineering, Ajou University, Suwon 16499, Korea

**Keywords:** tunnel field-effect transistor (TFET), heterojunction, band-to-band tunneling

## Abstract

The main challenge for tunnel field-effect transistors (TFETs) is achieving high on-current (*I*on) and low subthreshold swing (SS) with reasonable ambipolar characteristics. In order to address these challenges, Ge-channel heterostructure TFET with Si source and drain region is proposed, and its electrical characteristics are compared to other TFET structures. From two-dimensional (2-D) device simulation results, it is confirmed that the Si/Ge heterostructure source junction improves *I*on and SS characteristics by using the direct band-to-band tunneling current. Furthermore, the proposed structure shows suppressed ambipolar behavior since the Ge/Si heterostructure is used at the drain junction.

## 1. Introduction

Metal-oxide-semiconductor field-effect transistors (MOSFETs) have been consistently scaled down to the nanoscale, and power consumption (i.e., power density) is becoming an important concern to maintain Moore’s Law [1]. However, it is increasingly difficult to reduce operating voltage (*V*_DD_) while maintaining high on-off current ratio (*I*on/*I*off), since there is a fundamental limit of 60 mV/dec subthreshold swing (SS) at room temperature for MOSFETs [2,3]. This is due in part to gate-to-channel coupling limits, which cannot exceed unity due to voltage drop in the gate dielectric, as well as to the carrier injection mechanism, i.e., thermionic emission, which inevitably depends on the Boltzmann statistic [4]. Strategies to achieve SS < 60 mV/dec can be categorized in two ways. For example, negative capacitance field-effect transistor (NCFET) [5,6,7], resistive gate FET (ReFET) [8] and nano-electro mechanical FET (NEMFET) [9,10] mainly focus on the way to improve gate-to-channel coupling (decrease body factor (*m*) less than 1) with the help of novel gate stacks, while positive feedback FETs [11,12], impact ionization metal-oxide-semiconductor (I-MOS) [13,14] and tunnel FET (TFET) [15,16,17,18] try to change carrier injection mechanism by adopting novel operation methods. Among them, TFETs have received extensive research attention due to their high complementary MOS (CMOS) compatibility and scalability [19,20]. Although TFETs have raised the possibilities that they could succeed MOSFETs, they suffer various technical problems, such as low *I*on and disappointing SS. Heterojunction structures, which enable narrower local band-to-band tunneling (BTBT) barrier width (*W*_TUN_) by adopting narrow bandgap material like Ge, have been regarded as a powerful solution to address both problems simultaneously [21,22,23,24,25,26]. To the best of our knowledge, all previous heterostructure TFET used Ge at the source region. However, *I*on remains significantly behind the requirement for state-of-the-art technology and such device fabrication is not under consideration. 

This paper proposes a novel Ge channel heterostructure TFET and shows that the device achieves higher *I*on, lower SS, and reasonable ambipolar current (*I*_AMB_) using technology computer-aided design (TCAD) simulations. The proposed approach is straightforward: The Si-Ge heterojunction TFET differs from previous proposed systems by adopting Ge at channel not for source. Starting with systematic comparison among other heterojunction and homojunction TFETs’ current-voltage (*I-V*) characteristics, the proposed TFET’s operation mechanism is rigorously investigated to analyze its superior performance compared to others.

**Figure 1:**
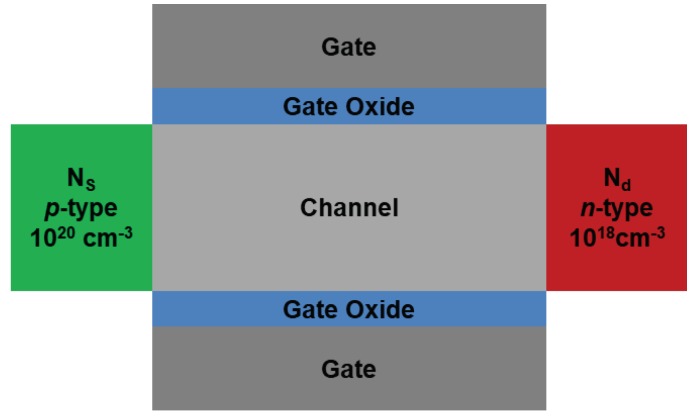
Basic schematic of tunnel field-effect transistor (TFET) structure in this study. In order to analyze the performance and the characteristics of the TFETs with two-dimensional device simulation, this kind of double gate structure is used representing the cross-section of the horizontal fin field-effect transistor (FinFET) structure.

Since the proposed approach is based on the change of channel material, a double gate structure which is highly compatible to the current logic device is used for an analysis (Figure 1). The detailed parameters used in this paper are listed below. In order to exclude short channel effect, physical gate length (*L*_G_) and body thickness (*T*_B_) between two gates are set at 100 and 20 nm, respectively. For the gate stack, gate oxide with 2 nm equivalent oxide thickness (EOT) and gate contact with 4.05 eV work function (*W*_FN_) are used, corresponding to highly doped *n*-type polysilicon. Source and channel doping concentrations are *p*-type 10^20^ cm^−3^ and undoped, respectively, to improve BTBT efficiency by suppressing degeneracy effect [27] while *n*-type 10^18^ cm^−3^ is used for draining to suppress an ambipolar behavior [28]. The feasibility of proposed TFET for high Ion and steep SS is compared with other TFETs by changing source/channel/drain material combinations, as summarized in Table 1. Considering a simple fabrication process using self-aligned epitaxy, a symmetric device structure is preferred. Therefore, the same materials are selected for the source and drain regions.

In order to analyze the electrical characteristics of the TFETs, two-dimensional (2-D) device simulations are carried out using Synopsys Sentaurus^TM^ (Ver. K-2015.06-SP1, Synopsys, Mountain View, CA, USA) [29]. Fermi–Dirac statistics, drift-diffusion carrier transport, Shockley–Read–Hall (SRH) recombination, modified local density approximation (MLDA), and dynamic non-local BTBT models are applied to accurately define device characteristics. Gate leakage current is neglected. The bandgap narrowing model is employed, since the source region is highly doped. Minimum conduction bands of *Γ*-valley and *L*-valley of Si and Ge are considered simultaneously. In case of the heterostructure, defects at Si and Ge interfaces are difficult to avoid and can significantly degrade device performance. However, in this research, these defects are neglected since the focus of this manuscript is mainly to optimize the TFET material combination under ideal conditions. For the calculation of BTBT generation rate (G) per unit volume in uniform electric field, Kane’s model is used as follows:

(1) $$G=A{\left(\frac{F}{F_{0}}\right)}^{P}\exp(-\frac{B}{F})$$

where *F* is electric field, and *F*_0_ = 1 V/cm; *P* = 2 and 2.5 for the direct and indirect tunneling, respectively. Pre-factor *A* and exponential factor *B* parameters for Si and Ge are calibrated by referring [30]. Although Kane’s model has some limitations, such as incorrectness in the presence of nonuniform fields and overestimation of the direct tunneling current [31,32,33], this model remains extensively used for TFET simulations.

## 2. Result and Discussion

**Figure 2:**
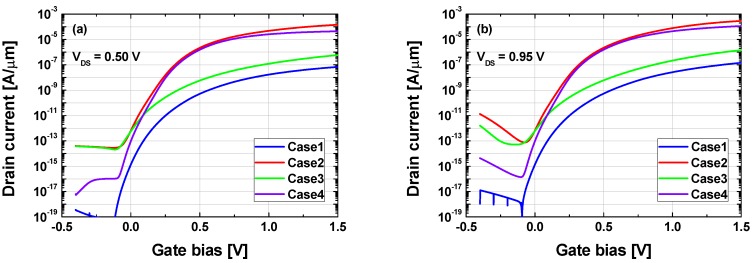

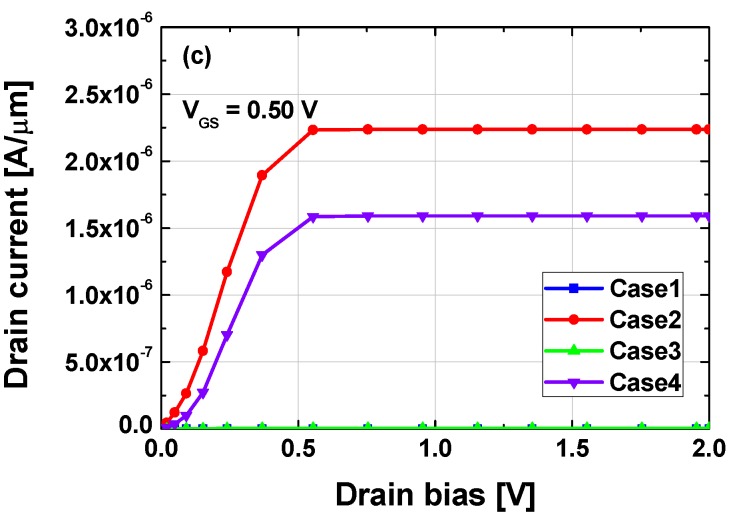
Simulated transfer characteristics ((**a**) *V*_DS_ = 0.5 V, (**b**) *V*_DS_ = 0.95 V) and output characteristics ((**c**) *V*_GS_ = 0.5 V) of Cases1–4.

**Figure 3:**
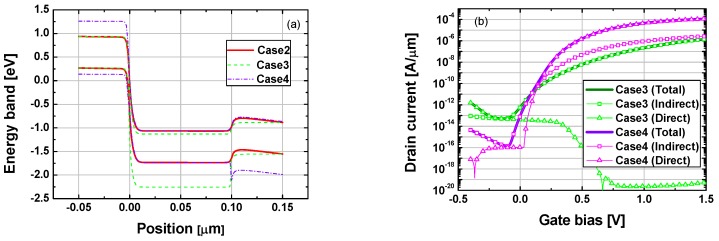

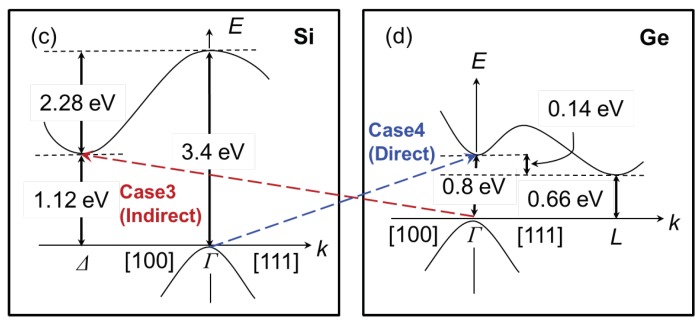
(**a**) Energy band diagrams for comparing Case2–Case4 at off-state (*V*_GS_ = 1.5 V, *V*_DS_ = 0.95 V). (**b**) Indirect tunneling, direct tunneling and total *I*_D_ of Case3 and Case4 structure. (**c**) Schematic energy band of Si. (**d**) Schematic energy band of Ge.

Simulated transfer characteristics for Case1–Case4 with 0.5 V or 0.95 V-drain voltage (*V*_DS_) are shown in Figure 2a,b. The drain voltage conditions were selected since they have been widely used in previous TFET studies [24,25,34]. The output characteristics of Case1–Case4 with 0.5 V V-gate voltage (*V*_GS_) are shown in Figure 2c. As shown in these figures, Si homojunction TFET (Case1) exhibited poor *I*on and SS characteristics, attributed in part to the large tunneling resistance due to the relatively large bandgap (~1.12 eV for Si), and in part to poor BTBT efficiency since Si has an indirect bandgap. In contrast, Ge homojunction TFET (Case2) showed significant *I*on and SS improvement, due to the narrower Ge bandgap (~0.67 eV). In addition, Krichnamohan et al. [24] previously showed that Ge can be regarded as having a pseudo-direct bandgap since its conduction band minimum at *Γ*-valley was only ~0.13 eV higher than that for the *L*-valley, as shown in Figure 3d. Consequently, BTBT probability is significantly increased and results in up to several hundred μA/μm-*I*on. However, Case2 suffered from an increased *I*_AMB_ since the BTBT resistance at the drain junction was also reduced. 

Ge/Si/(Ge) heterojunction (Case3) has been regarded as one of the most promising structures to compromise *I*on and *I*off. However, *I*on <1 μA/μm requires further improvement to provide reasonable operating speed and *I*_AMB_ should be significantly reduced. Although Case3 can operate as much as Case2, adopting Ge channel-to-gate overlap region, it requires advanced process capability [28,35].

Proposed TFET (i.e., Case4: Si source and Ge channel) showed better *I*on and SS characteristics than Case3 (Ge source and Si channel) structure, as can be seen in Figure 2a,b. It was thoroughly deviated from the general expectations as both *W*_TUN_ and the tunnel window (i.e., difference between valence band maximum at source and conduction band minimum at channel) of Case4 were larger and smaller than that for Case3, respectively (Figure 3a). On the other hand, Case4 showed similar *I*on and SS characteristics to Case2 having Ge homojunction between the source and the channel region. To the best of our knowledge, this is the first report regarding this characteristic for Case4 type structures.

To investigate the reasons of the remarkable transfer characteristics in Case4 structure, tunneling currents in Case3 and Case4 were divided into indirect and direct components, as shown in Figure 3b. For Case3, indirect BTBT dominated total *I*_D_ and the direct component was negligible when gate voltage (*V*_GS_) was higher than 0 V. In contrast, the transfer characteristic of Case4 showed quite a different trend from that of Case3 structure. When *V*_GS_ = 0 V, the indirect tunneling current was much higher than the direct one. However, as *V*_GS_ increased, direct BTBT exceeded indirect and total *I_D_*, and SS characteristics for Case4 were mainly determined by the direct tunneling current. These results can be further analyzed by the energy band structure at the junction between source and channel. Schematic energy band diagrams of Si and Ge are shown in Figure 3c,d, respectively. When on-current flows in Case3, electrons from the valence band of the Ge source are injected into the conduction band of the Si channel. In contrast, in on-state, electrons in Case4 transfer from the valence band of the Si source to the conduction band of the Ge channel. Therefore, direct BTBT for Case3 barely occurs since the energy level of the *Γ* valley is 2.28 eV higher than the *Δ* valley at the conduction band of Si. On the other hand, in case of Si to Ge tunneling junction of Case4 structure, the energy difference between the *Γ* valley and the *L* valley at the conduction band of Ge is only 0.14 eV. Hence direct BTBT current can significantly contribute to total *I*_D_ as *V*_GS_ increases.

**Figure 4:**
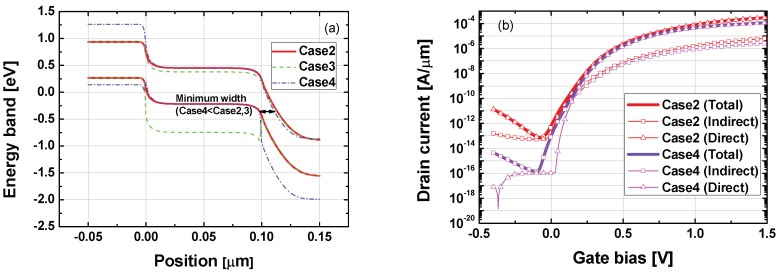
(**a**) Energy band diagrams for comparing Case2–Case4 at off-state (*V*_GS_ = −0.4 V, *V*_DS_ = 0.95 V). (**b**) Indirect tunneling, direct tunneling and total drain current of Case2 and Case4 structures.

As well as improved *I*on and SS characteristics, Case4 (Ge channel and Si drain) structure also exhibited superior ambipolar characteristics compared to Case2 (Ge homojunction) and Case3 structures, as shown in Figure 2. The energy band diagrams for Case2–Case4 structures at off-state (*V*_GS_ = −0.4 V and *V*_DS_ = 0.95 V) are shown in Figure 4a, to identify the reason for reduced *I*_AMB_ in Case4 structure. Interestingly, Case4 structure had minimum *W*_TUN_, in contrast to the smallest *I*_AMB_ in Figure 3b. The main reason for these improved ambipolar characteristics was the effective suppression of direct BTBT components. Direct tunneling leakage of Case4 structure was negligible, confirming Case4 structure’s significantly improved ambipolar characteristics, as seen in Figure 4b.

The electrical characteristics of Case1–Case4 structures are summarized in Table 2. The SS was extracted at 0.95 V-*V*_DS_ and 0 V-*V*_GS._ The *I*on and *I*off were defined as *I*_D_ with 0.95 V-*V*_DS_ at 1.5 V-*V*_GS_ and 0 V-*V*_GS_, respectively. As discussed above, Case4 exhibited superior *I*on/*I*off among the simulated structures, achieving the highest *I*on due to direct tunneling at the source junction, and effectively suppressing ambipolar characteristics by reducing direct tunneling leakage current at the drain region. However, the advantages of Case4 structure are limited to *n*-type TFETs, with *p*-type TFET exhibiting dominant indirect tunneling with the Ge/Si heterostructure at the source junction.

To fabricate the Si/Ge heterostructure, the Ge layer should be grown by epitaxial processes [23]. Since Ge is confined in the channel region for Case4 structure, this layer can be easily fabricated using self-aligned epitaxial process during the replacement-metal-gate (RMG) process [36]. Ge condensation technique can be applied to implement the high Ge content SiGe channel close to pure Ge [37]. After SiGe layer growth around the channel layer in the RMG process step, the enriched Ge channel layer can be formed by selective Si oxidation and Ge diffusion from the initial grown SiGe layer. In addition, since Si is used for the source region, it is relatively easy to achieve high doping compared to when using Ge.

## 3. Summary

In this research, the appropriate material combination for source/channel/drain regions in TFET was investigated to improve electrical characteristics. From the device simulation results, it was verified that the Si/Ge/Si combination exhibits outstanding *I*on and SS characteristics, using direct BTBT of the Si/Ge heterostructure at the source junction. Additionally, ambipolar effect, one of the critical disadvantages of TFETs, could be suppressed by increasing tunneling resistance at the drain junction with the Ge/Si heterostructure. From the perspective of process complexity, the proposed structure can be easily fabricated using simple self-aligned epitaxy as the same material (Si) is used for the source and drain region.

## Figures and Tables

**Table 1:** Material combinations of the devices analyzed in this study.

Case	Source (*p*-type 10^20^ cm^−3^)	Channel (Undoped)	Drain (*n*-type 10^18^ cm^−3^)
Case1	Si	Si	Si
Case2	Ge	Ge	Ge
Case3	Ge	Si	Ge
Case4	Si	Ge	Si

**Table 2:** Comparison of the electrical characteristics of Case1–Case4 structures.

Case	Subthreshold Swing (SS) [mV/dec]	*I*_on_ [μA/μm]	*I*_off_ [pA/μm]	*I*_on_/*I*_off_
Case1	40.5	0.1	0.001	1.0 × 10^8^
Case2	45.3	294.5	0.666	4.4 × 10^8^
Case3	68.4	1.5	0.602	2.5 × 10^6^
Case4	35.8	115.1	0.095	1.2 × 10^9^
